# Development and validation of a patient-tailored dose regime in myocardial perfusion imaging using conventional SPECT

**DOI:** 10.1007/s12350-015-0246-9

**Published:** 2015-09-09

**Authors:** J. D. van Dijk, P. L. Jager, J. P. Ottervanger, J. de Boer, A. H. J. Oostdijk, E. M. Engbers, C. H. Slump, S. Knollema, J. A. van Dalen

**Affiliations:** Department of Nuclear Medicine, Isala Hospital, PO Box 10400, Zwolle, 8000 GK The Netherlands; Department of Cardiology, Isala Hospital, Zwolle, The Netherlands; MIRA Institute for Biomedical Technology and Technical Medicine, University of Twente, Enschede, The Netherlands; Department of Medical Physics, Isala Hospital, Zwolle, The Netherlands

**Keywords:** SPECT, myocardial perfusion imaging, activity, dose

## Abstract

**Background:**

The decreasing image quality in heavier patients can be compensated by administration of a patient-specific dose in myocardial perfusion imaging (MPI) using a cadmium zinc telluride-based SPECT camera. Our aim was to determine if the same can be achieved when using a conventional SPECT camera.

**Methods:**

148 patients underwent SPECT stress MPI using a fixed Tc-99m tetrofosmin tracer dose. Measured photon counts were normalized to administered tracer dose and scan time and were correlated with body weight, body mass index, and mass per length to find the best predicting parameter. From these data, a protocol to provide constant image quality was derived, and subsequently validated in 125 new patients.

**Results:**

Body weight was found to be the best predicting parameter for image quality and was used to derive a new dose formula; *A*_admin_ (MBq) = 223·body weight (kg)^0.65^/*T*_scan_ (min). The measured photon counts decreased in heavier patients when using a fixed dose (*P* < .01) but this was no longer observed after applying a body-weight-dependent protocol (*P* = .20).

**Conclusions:**

Application of a patient-specific protocol resulted in an image quality less depending on patient’s weight. The results are most likely independent of the type of SPECT camera used, and, hence, adoption of patient-specific dose and scan time protocols is recommended.

## Introduction

Myocardial perfusion imaging (MPI) using single-photon emission computed tomography (SPECT) is one of the most validated non-invasive methods to test for ischemia in patients suspected of stable coronary artery disease.^1^ Remarkably, the dosages to administer vary widely between institutions, and in most European countries, fixed tracer doses are recommended.^2^ Yet, a decreasing image quality is observed in heavier patients in clinical practice. The European (EANM/ESC) guidelines still recommend a fixed tracer dose,^2^ whereas the American (ASNC) guidelines propose to adjust the dose upward for patients heavier than 70 kg by 11.5 MBq/kg for Tc-99m.^3^ However, no references for this adjustment are provided, and possible differentiation to account for a different scanner set-up or sensitivities is not mentioned.

Several studies have demonstrated a decrease in measured photon counts for increasing body weights.^4^-^7^ They propose different correction formulas to compensate for this decrease. Those methods were mainly focused on correcting for heavier patients, and only one correction formula was validated in clinical practice.^8^ This validation was only performed in patients weighing more than 100 kg, ignoring the possible benefit of a lower dose for leaner patients. Moreover, the influence on image quality by reader interpretation was not assessed.^8^

We recently developed a method to derive a body-weight-dependent tracer dose or scan time protocol. Application led to a constant image quality in MPI using a gamma camera equipped with cadmium zinc telluride (CZT) detectors.^9^ This validated protocol seems not directly applicable on conventional gamma cameras, equipped with sodium iodide crystals, due to their lower detector sensitivity, image contrast, and different detector configuration.^10^-^12^ Hence, the aim of this study was to test if introducing a patient-specific dose and scan time protocol for MPI SPECT using a conventional camera results in an image quality independent of patients’ physical characteristics.

## Materials and Methods

In this study, we used a comparable methodology as previously described by van Dijk et al.^9^ All patients were scanned according to the standard clinical protocol valid at the time of acquisition. Based on the outcomes of the tracer dose and scan time deriving part of this study, the clinical protocol was changed in the hospital. For this reason, approval by the medical ethics committee was not required. All patients provided written informed consent for the use of their data for research purposes.

### Study Population

We retrospectively included a total of 273 consecutive patients who underwent clinically indicated SPECT stress MPI on a conventional dual-detector gamma camera (Ventri, GE Healthcare). All patients underwent a stress-first 1-day Tc-99m tetrofosmin SPECT protocol. The first 105 patients were included in the dose and scan time deriving part of this study (further referred to as group A). To obtain a patient population with a sufficient amount of patients in the full range of body weights that are encountered in clinical practice, another 43 patients were specifically added such that at least 10 patients fell into one of the following body weight categories: <60, 60-70, 71-80, 81-90, 91-100, 101-110, 111-120, 121-130, and >130 kg. Moreover, additional 99 patients were consecutively included for the validation part of this study, and another 26 patients were added to obtain a similar amount of patients in each body weight category as in group A (further referred to as group B).

### Patient Preparation and Image Acquisition

Patients were requested not to use any nicotine- or caffeine-containing beverages for 24 hours and to discontinue persantin for 48 hours prior to scanning. Pharmacological stress was induced by intravenous adenosine (140 μg/kg/min for 6 minutes) or regadenoson (5 mL with 400 μg for 15 seconds followed by a saline flush). Only pharmacologic stress was used due to logistic reasons, in particular the high patient throughput in our center.^13^ A fixed tracer dose of 370 MBq Tc-99m tetrofosmin (500 MBq for patients with a body weight of more than 100 kg) was administered intravenously at peak stress in group A.

Patients were requested to consume at least half a chocolate bar and drink three cups of water post-injection to reduce sub-diaphragmatic activity uptake and improve image quality of the inferior wall. Stress imaging was performed 45-60 minutes post-injection. All patients were scanned in supine position, with their arms placed above their heads using a fixed scan time of 10 minutes. Images were acquired using a double detector, low-energy high-resolution collimator, with an elliptical orbit with step-and-shoot acquisition at 6° intervals over a 180° arc (45° anterior oblique to 45° left posterior oblique) with 15 views (40 seconds per view), and a 64 × 64 matrix using a 20% symmetrical energy window centered at 140 keV.

Subsequently, the emission images were reconstructed by applying an iterative dedicated reconstruction algorithm with maximum-likelihood expectation maximization (Myovation, GE Healthcare). Each image was automatically normalized to the maximum peak activity, and the segmental uptake values were presented as the percentage of the maximum myocardial regional uptake. The images were displayed in the traditional short, vertical long, and horizontal long axes and reviewed using a color scale.

### Deriving a Patient-Specific Tracer Dose and Scan Time Protocol

**Figure 1:**
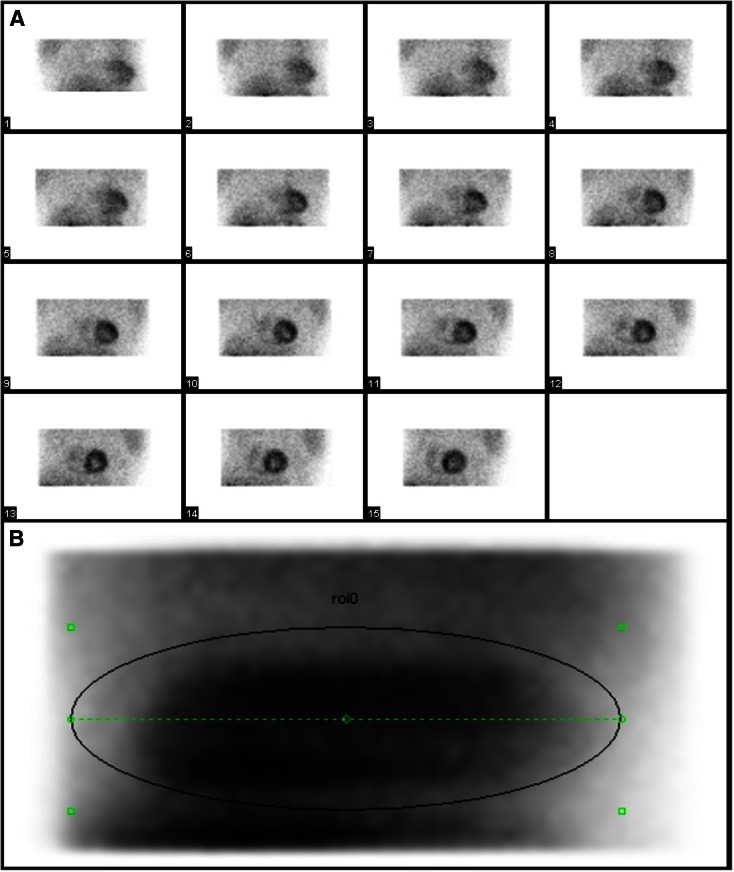
An example of (**A**) 15 of the 30 raw-emission images covering the myocardial region and (**B**) the image showing the summation of all 30 raw-emission images including the manually drawn elliptical region of interest covering all myocardium positions to determine the measured photon counts

First, the total number of measured photon counts in the myocardial region was determined in the raw-emission data for each scan. This was done by summing the 30 (2 detectors × 15 views) raw-emission images into one image and consecutively manually drawing an elliptical region of interest covering all myocardium positions, as shown in Figure 1. Using elliptical regions allowed us to exclude surrounding activity and to include only the counts originating from the myocardial region.

Next, the measured photon counts were normalized to the product of the tracer dose corrected for radioactive decay and scan time, *T*_scan_ (min). To find the patient-specific parameter (*P*) best explaining the decrease in image quality for heavier patients, the normalized photon counts, *C*_norm_, were fitted to three patients’ physical characteristics: body weight, mass per length, and body mass index (BMI) using a power-law function:

1 $$ C_{\text{fit}} = a \cdot P^{b}. $$

Here, *a* and *b* are fit parameters. Parameter selection was based on practicality in use and power to correct for the varying image quality in MPI as previously demonstrated.^9^

When a decrease in the normalized photon counts can be explained by a patient-specific parameter, this allows making a correction using the tracer dose and/or scan time. Subsequently, this can be used to derive a protocol resulting in a constant number of counts (*C*), and accordingly a constant image quality, ideally independent of patients’ physical characteristics.^9^ For this study, *C* was set equal to the average number of photon counts measured in all scans. The following formula describes the relation between *C* and the recommended patient-specific scan time and/or tracer dose to be administered, *A*_admin_:

2 $$ C = C_{\text{fit}} \cdot \frac{{A_{\text{admin}} \cdot T_{\text{scan}} }}{K}. $$

Here, *K* is the correction factor for radioactive decay between administration of tracer dose and SPECT acquisition (1.12 for 60 minutes). A linear count rate response was assumed because the maximal count rate encountered in this study (6 kcounts/second) was much lower than the count rates associated with the occurrence of dead time effects in conventional cameras (>200 kcounts/second).^14^

When combining Eqs. 1 and 2, this results into

3 $$ A_{\text{admin}} = \frac{{C \cdot P^{ - b} \cdot K}}{{a \cdot T_{\text{scan}} }}. $$

Equation 3 shows that *A*_admin_ and *T*_scan_ are interchangeable, as suggested by Oddstig et al^15^ i.e., instead of introducing a patient-specific dose, a patient-specific scan time may be adopted. In addition, the dose to administer can be reduced, while increasing the scan time to obtain the same image quality up to certain limits due to possible patient motion.

### Validating the Derived Tracer Dose and Scan Time Protocol

The derived tracer dose and scan time formula, according to Eq. 3, was implemented in our routine clinical protocol.

The image quality for all 247 reconstructed myocardium images in both group A and B was scored by three independent experienced nuclear medicine physicians with overread in case of discordance by a fourth expert. A visual 4-point grading scale (1-poor, 2-fair, 3-good, and 4-excellent) was employed. The following parameters were considered: myocardial count density and uniformity in well-perfused areas, signal to background noise, and shape of the left ventricle. All readers were blinded for patient characteristics, and the images were presented in random order.

The image quality was compared between groups A and B. The correlation between the best explaining parameter and both the image quality and measured photon counts was assessed to determine if they were independent when applying a patient-specific protocol.

### Statistics

All patient-specific parameters and characteristics were determined as mean ± standard deviation (sd) and compared using the chi-square and unpaired *t*-tests using Stata software (StataSE 12.0). To test if the slope of *C*_fit_ differed significantly from zero for each patient-specific parameter, implying a significant correlation with *C*_norm_, *t*-tests were performed. Coefficients of determination (*R*^2^) were determined for all fits, and the fit errors were calculated for each data point using (*C*_fit_ − *C*_norm_)/*C*_fit_·100%. To determine if the fit results differed between the three selected parameters, the fit error distributions were compared using the *F*-test. Next, the patient-specific parameter best explaining the normalized photon counts was selected using the results of *R*^2^ and the *F*-tests. The correlation between the best explaining parameter and both the measured photon counts and image quality was calculated in both groups using the Pearson correlation coefficient and the Spearman’s rank correlation coefficient, respectively. In addition, the correlation between measured photon counts and image quality was calculated using the Spearman’s rank correlation coefficient. Furthermore, the influence of interpreted scan outcomes on the image quality was assessed using the chi-square test. The body weight for the patient scans interpreted as normal or abnormal was compared for both groups using a *t*-test.

The level of statistical significance was set to 0.05 for all statistical analyses.

## Results

The baseline characteristics of all included patients are summarized in Table 1.

**Table 1:** Baseline characteristics, interpreted scan outcomes, and administered tracer dose of all 273 patients who underwent clinically indicated MPI SPECT

Characteristic	Group A (n = 148)	Group B (n = 125)	*p* value (chi-square/*t*-test)
Age (years)	67.5 ± 10.8	68.5 ± 9.8	0.43
Male gender (%)	55.4	66.4	0.06
Body weight (kg)	85.1 ± 22.2	87.3 ± 22.1	0.45
Height (cm)	172 ± 10.2	174 ± 9.5	0.08
BMI (kg/m^2^)	28.6 ± 6.3	28.5 ± 5.8	0.91
Current smoking (%)	15.2	10.5	0.31
Hypertension (%)	62.3	57.0	0.52
Diabetes (%)	26.1	30.7	0.43
Dyslipidemia (%)	63.7	54.5	0.17
Family history (%)	66.4	56.9	0.09
Normal MPI scan (%)	34.3	38.2	0.60
Ischemic defect (%)	32.4	35.5	0.61
Non reversible defect (%)	44.5	40.9	0.59
Tracer dose at acquisition (MBq)	343 ± 70	352 ± 57	0.23

Data are presented as mean ± standard deviation or percentages

### Deriving a Patient-Specific Tracer Dose and Scan Time Protocol

The mean number of measured photon counts in the myocardial region in group A was 812 × 10^3^ ± 197 × 10^3^. This was 236 ± 62 counts·MBq^−1^·min^−1^ for the normalized photon counts.

**Figure 2:**
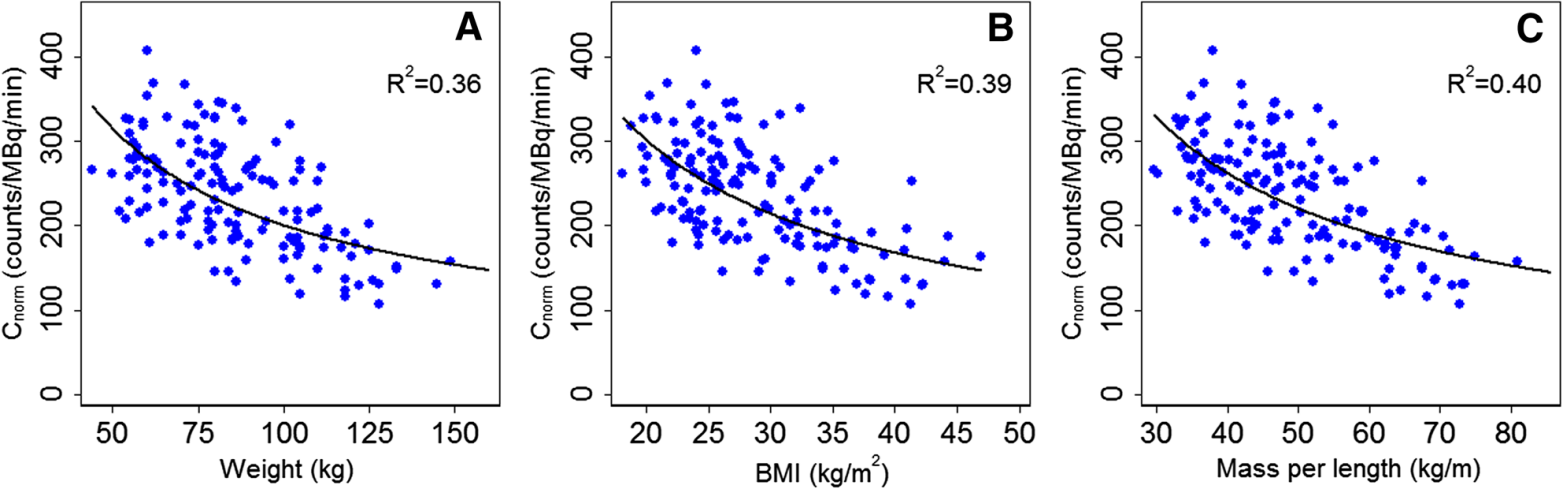
Measured photon counts normalized to tracer dose and scan time as a function of (**A**) body weight, (**B**) BMI, and (**C**) mass per length. The *solid lines* represent the power-law fits and the coefficients of determination are also shown for each fit

The slope of all three fits, describing the relation between the normalized photon counts and the three patient-specific parameters, was found to be statistically different from zero (*P* < .001), as illustrated in Figure 2. As hypothesized, the measured photon counts normalized to dose and scan time decreased for all patient-specific parameters, explaining the lower image quality encountered in heavier patients in clinical practice. The calculated fit parameters *a* and *b* and the coefficient of determination are shown in Table 2.

**Table 2:** Fit parameters describing the relation between the normalized photon counts and each patient-specific parameter, including the coefficients of determination (*R* ^2^) and result of the error distribution comparison

Parameter	*a* (95% CI)	*b* (95% CI)	*R* ^2^	*F*-test (*P* value)
Body weight	4063 (2174:7593)	−0.65 (−0.80:−0.51)	0.36	Reference
BMI	3812 (2160:6727)	−0.85 (−1.02:−0.68)	0.40	0.79
Mass per length	4666 (2547:8551)	−0.78 (−0.94:−0.62)	0.39	0.73

Body weight was chosen as the patient-specific parameter best explaining the normalized photon counts based on its practicality in use, a comparable standard deviation of the error distributions (*P* > .7) and *R*^2^ value, and was further used in this study.

**Figure 3:**
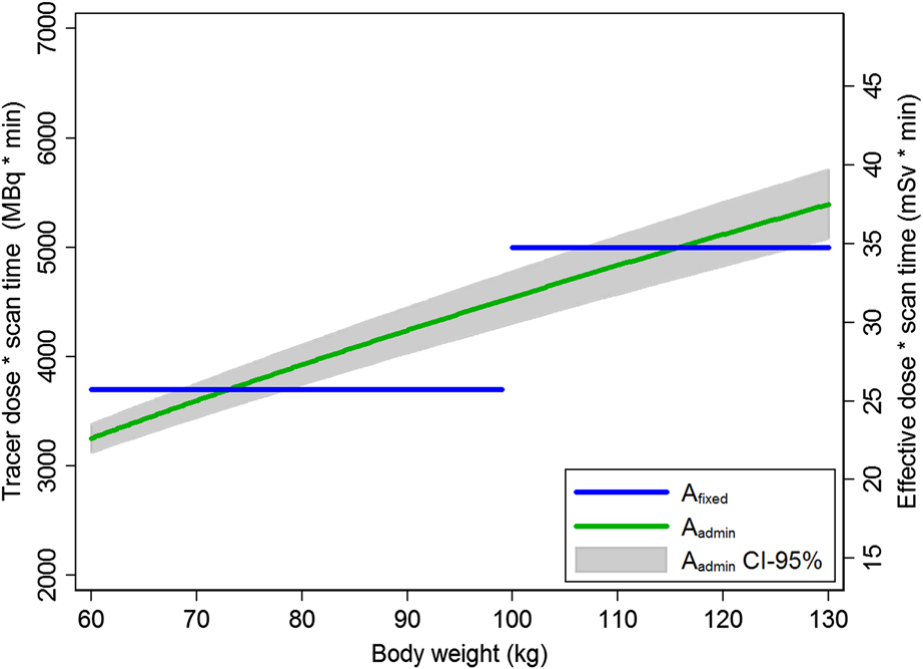
Line graph demonstrating the previously applied fixed Tc-99m tracer dose and scan time product (A_fixed_) and the derived body-weight-dependent patient-specific protocol (see Eq. 3) as applied in group B (A_admin_), including the 95% confidence interval. The right *y*-axis shows the product of the effective patient dose and scan time

Using Eq. 3, a body-weight-dependent patient-specific dose and scan time protocol was derived and consecutively validated. The protocol can be described by *A*_admin_ (MBq) = 223·body weight (kg)^0.65^/*T*_scan_ (min) or *A*_admin_ (mCi) = 6.0·body weight (kg)^0.65^/*T*_scan_ (min), and is illustrated in Figure 3 and shown in Table 3. This formula is based on patients with body weights ranging between 60 and 130 kg, and hence, the tracer dose ranged from 319 to 564 MBq.

**Table 3:** Derived body-weight depending Tc-99m tetrofosmin tracer dose when using a fixed scan time of 10 minutes in stress MPI using a conventional SPECT camera

Body weight (kg)	Tracer dose	
	(MBq)	(mCi)
<60	319	8.6
65	347	9.4
70	366	9.9
75	384	10.4
80	402	10.9
85	419	11.3
90	436	11.8
95	453	12.2
100	470	12.7
105	486	13.1
110	502	13.6
115	518	14.0
120	534	14.4
125	549	14.8
130	564	15.3
>130	564	15.3

### Validating the Derived Tracer Dose and Scan Time Protocol

The mean number of measured photon counts in the myocardial region in group B was 947 × 10^3^ ± 188 × 10^3^. This was 276 ± 71 counts·MBq^−1^·min^−1^ for the normalized photon counts.

**Figure 4:**
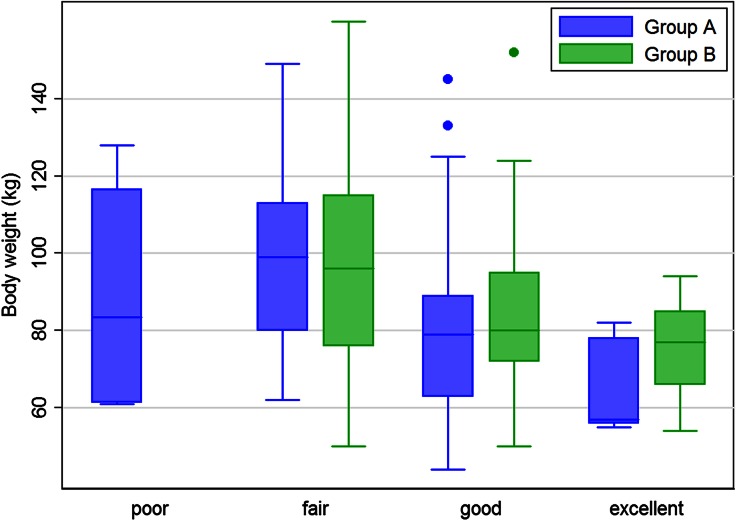
Boxplot showing the relation between body weight and scored image quality for both patient groups. A significant relation was found for group A (fixed tracer dose, *P* = .01) and group B (body-weight depending tracer dose, *P* = .03)

**Figure 5:**
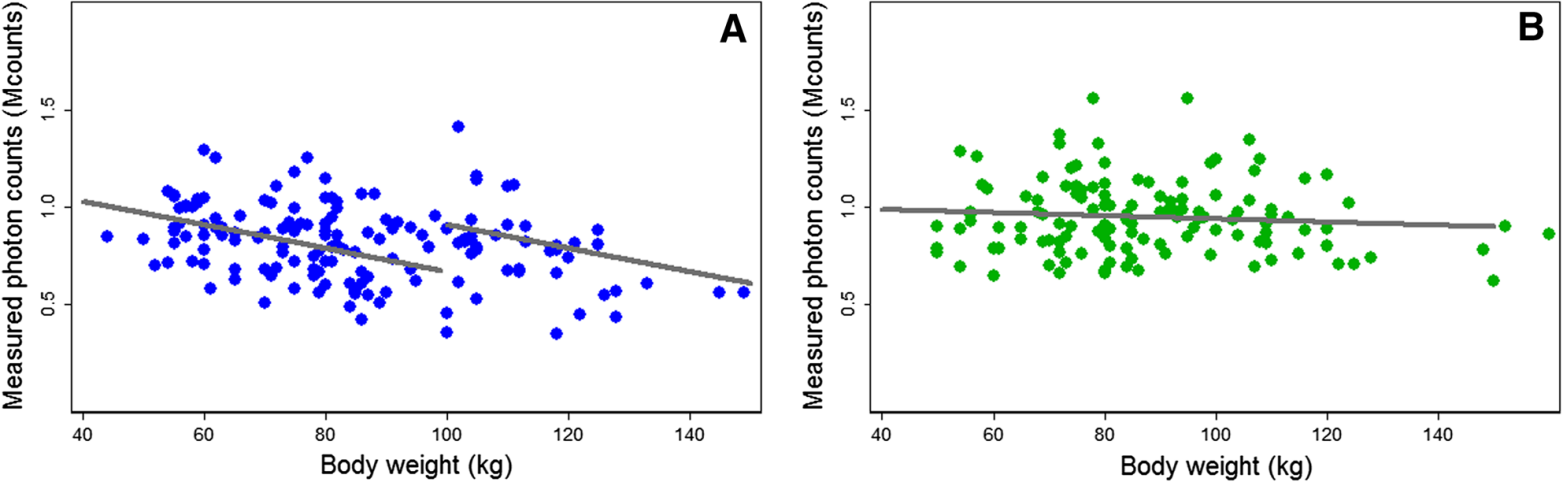
Measured photon counts as a function of body weight for (**A**) group A (fixed dose) and (**B**) group B (body weight depending protocol). The *lines* represent the linear fits where in (**A**) the two fits correspond to patients weighing less and more than 100 kg. The slope of the fit did significantly differ from zero in group A (*P* < .001), whereas the measured photon counts became independent of body weight after applying the new protocol (*P* = .29)

Within the set of scored images, 3% and 0% were scored as poor, 31% and 33% as fair, 63% and 59% as good, and 3% and 8% as excellent in group A and B, respectively. The interpreted image quality decreased for heavier patients in both group A (*P* < .001) and group B (*P* = .003), as illustrated in Figure 4. However, different correlations between the measured photon counts and body weight were observed between both groups, as illustrated in Figure 5. A significant correlation between counts and body weight was observed in group A (*P* < .001), whereas this correlation was absent in group B (*P* = .29). Moreover, a significant correlation was found between measured photon counts and image quality in both group A and B (*P* = .01 and .03, respectively).

Patients with scans interpreted as normal had a significant higher image quality in both groups (*P* < .003). In addition, patients without a normal scan were significantly heavier in group B (*P* = .02), whereas this was not the case in group A (*P* = .18).

## Discussion

In the present study, we derived and subsequently validated a body-weight depending tracer dose and scan time protocol to obtain a more constant image quality independent of patients’ physical characteristics in MPI using SPECT. Although a significant decreasing image quality was observed for heavier patients in both group A and B, the measured photon counts became independent of body weight after applying the new protocol. Hence, we can assume that a patient-specific protocol in MPI using a conventional SPECT camera results in a more consistent image quality less dependent of patient’s physical characteristics.

**Figure 6:**
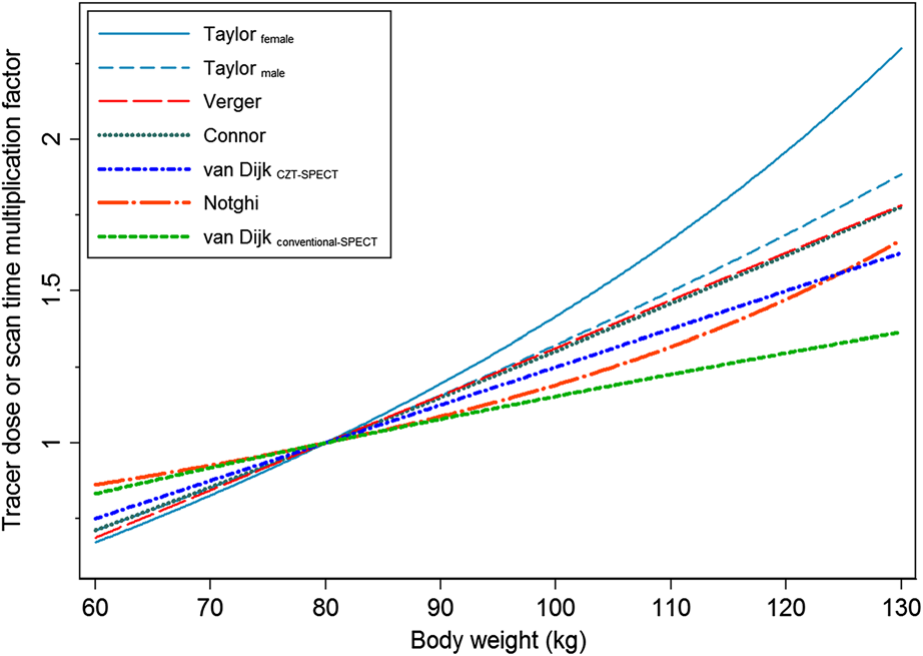
Differences in the proposed correction factors to adjust the tracer dose or scan time for leaner and heavier patients, normalized to an average patient of 80 kg. Included studies are Taylor et al differentiating between males and females,^6^ Verger et al,^7^ O’Connor et al,^4^ van Dijk et al (CZT-SPECT),^9^ Notghi et al,^5^ and the present study (van Dijk et al, conventional SPECT)

Similar nonlinear relations between normalized photon counts and body weight as found in this study were observed previously.^4^-^7^,^9^ Yet, the derived formulas to correct for the decreasing photon counts in heavier patients vary. After normalizing these formulas to an average patient of 80 kg, the correction factors to adjust the tracer dose for a 130 kg patient vary between 1.4 and 2.3, as illustrated in Figure 6. These differences are most likely due to the variation in methodologies used to estimate the photon counts originating from the myocardium. The studies which measured the photon counts in the raw-emission images, to eliminate possible influences of reconstruction algorithms, all propose correction factors varying between 1.4 and 1.6 for a 130 kg patient.^5^,^9^ However, higher correction factors are proposed by the studies which estimated the counts in a region of interest in the reconstructed images, resulting in correction factors varying between 1.7 and 2.3.^4^-^7^ Yet, a part of the variation in correction formulas might also be due to the exclusion of patients, for example, patients with perfusion defects,^5^ three vessel disease,^6^ or who underwent rest imaging.^7^ Only three studies, including the present study, are known to have validated their correction formula in clinical practice.^8^,^9^ Notghi et al and van Dijk et al also demonstrated to obtain a constant image quality independent of patients’ physical characteristics. Yet, we are the first to demonstrate that a patient-specific formula is also applicable in patients weighing less than 100 kg when using a conventional SPECT camera.^8^ Moreover, it seems that the higher correction factors as derived and proposed in the non-validated studies, as illustrated in Figure 6, are not necessary.

There is a large variation in the correction formulas as proposed by our study and the other two validated studies. These differences might be explained by the use of different fits to describe the relation between image quality and body weight, as shown for the present study in Figure 2. Notghi et al used a linear fit instead of power-law fit, despite a similar distribution.^5^ This resulted in relatively large differences in the multiplication factor for heavy patients, as illustrated in Figure 6. Moreover, they derived their formula in patients weighing less than 110 kg. Extrapolation of this formula might therefore result in inaccurate corrections for patients over 110 kg. The differences in correction formulas might also be due to different scanner specifications, such as collimator design, geometrical detector configuration, and moving or stationary detectors. This could explain the difference in correction formula between the present study and the study performed on a CZT-SPECT scanner using a comparable methodology.^9^

The current study has some limitations. First, a decreasing image quality for heavier patients was still found in group B, possibly indicating the limited effect of applying the new protocol. Nevertheless, the measured photon counts became independent of patients’ size after applying the new protocol. Moreover, the significant relation between image quality and photon counts suggests that a constant number of photon counts result in a constant image quality, as previously demonstrated using a CZT-based camera.^9^ The still existing relation between image quality and weight in group B might be due to readers being susceptible for perfusion defects in MPI SPECT. Patient scans with perfusion defects had a significantly lower image quality, and these patients were also significantly heavier in group B, possibly explaining the decreasing image quality for heavier patients.^16^,^17^ Second, the proposed formula corrects for the varying image quality but does not represent the minimal tracer dose to administer to obtain an accurate diagnostic image quality, as previously performed on a CZT-camera.^18^,^19^ The proposed formula was based on the average photon counts measured in group A. Hence, the mean radiation exposure was comparable between group A and B, whereas the radiation justification improved in group B, as illustrated in Figure 3. Moreover, four (3%) images were scored as poor in group A vs none in group B, and only five (3%) and ten (8%), respectively, were scored as excellent. This might indicate that increasing or decreasing the tracer dose and scan time product would not be necessary. It would lead to either an unacceptable amount of poor images or many excellent images, which is considered undesirable. Third, the tracer doses mentioned in this study are absolute activities including possible residual activities in the syringes. An internal study revealed that 10.6% ± 1.2% Tc-99m tetrofosmin remained in the syringe after administration. The small variation indicates the limited influence on our results. Finally, the variation in photon counts between patients with comparable body weights is still relatively large in group B, as observed in previous studies.^4^-^7^,^9^ This might be due to the large variation in Tc-99m tetrofosmin uptake,^16^,^17^ influence of gastro-intestinal activity despite the cardio-specific regions of interests, different body compositions such as varying tissue layer between heart and scanner or size of the myocardium, heart function, liver excretion, and/or gender differences.^6^,^9^

Our present findings demonstrate the added value of applying patient-specific dose and scan time protocols in clinical practice for a conventional SPECT camera. This protocol can be adopted using different settings and/or type of SPECT camera by multiplying the currently applied tracer dose and scan time product for an average patient of 80 kg by the correction factor as shown in Figure 6. This formula is based on patients with body weights ranging between 60 and 130 kg. Therefore, caution should be taken by extrapolating this formula to patients outside this body weight range. The patient population we encounter in clinical practice might not be fully representative for other institutions encountering even heavier patients with higher BMIs. However, we could not include a sufficient number of patients heavier than 130 kg to be able to further extrapolate the protocol for these patients. Moreover, when clinically implementing this formula, it is advised to validate whether the proposed correction formula holds true for the applied settings and type of scanner used.

## Conclusions

The use of a fixed tracer dose of Tc-99m tetrofosmin in MPI using a conventional SPECT camera results in a decreasing image quality in heavier patients. Application of the derived patient-specific tracer dose and scan time protocol, *A*_admin_ (MBq) = 223·body weight (kg)^0.65^/*T*_scan_ (min), resulted in an image quality less dependent on weight. This provides a better radiation exposure justification. The results are most likely independent of the type of SPECT camera used, and, hence, adoption of patient-specific dose and scan time protocols is recommended.

## New Knowledge Gained

A constant image quality can be obtained in MPI using conventional SPECT by application of a body-weight-dependent activity and scan time protocol. This protocol corrects for the higher photon attenuation in heavier patients. It results in a number of measured photon counts which is independent of patients’ size. This study shows good agreement with the study on a CZT-camera, making these observations applicable to all SPECT cameras.

## Abbreviations

MPI: Myocardial perfusion imaging
SPECT: Single-photon emission computed tomography
CZT: Cadmium zinc telluride
BMI: Body mass index
